# Sodium Silicate from Rice Husk Ash and Their Effects as Geopolymer Cement

**DOI:** 10.3390/polym14142920

**Published:** 2022-07-19

**Authors:** Lia Handayani, Sri Aprilia, Abdullah, Cut Rahmawati, Teuku Budi Aulia, Péter Ludvig, Jawad Ahmad

**Affiliations:** 1 Faculty of Fisheries, Universitas Abulyatama, Aceh Besar 23372, Indonesia; liahandayani_thp@abulyatama.ac.id; 2 Geopolymer and Nanomaterials Research Center, Universitas Abulyatama, Aceh Besar 23372, Indonesia; 3 Department of Chemical Engineering, Engineering Faculty, University of Syiah Kuala, Banda Aceh 23111, Indonesia; sriaprilia@unsyiah.ac.id; 4 Department of Civil Engineering, Engineering Faculty, University of Syiah Kuala, Banda Aceh 23111, Indonesia; abdullahmahmud@unsyiah.ac.id (A.); aulia@unsyiah.ac.id (T.B.A.); 5 Department of Civil Engineering, Universitas Abulyatama, Aceh Besar 23372, Indonesia; 6 Department of Civil Engineering, Federal Center for Technological Education of Minas Gerais, Belo Horizonte 30421-169, Brazil; peter@cefetmg.br; 7 Department of Civil Engineering, Military College of Engineering, NUST, Risalpur 24080, Pakistan; jawadcivil13@scetwah.edu.pk

**Keywords:** sodium silicate, activator, geopolymer cement, rice husk ash, fracture toughness

## Abstract

Sodium silicate is a commonly used activator in geopolymer that is produced commercially. In this study, rice husk ash (RHA) from agricultural waste was used to synthesize sodium silicate as an activator for geopolymer cement. This white ash was applied for producing sodium silicate with different molarities (8, 10, and 12) and then used to synthesize fly ash-based geopolymer cement. Scanning Electron Microscopy (SEM), X-ray Diffraction (XRD), and Fourier Transform Infrared Spectroscopy (FTIR) were applied to investigate the micro-characteristics of the geopolymerization products. Bulk density, water absorption, compressive strength, flexural strength, and fracture toughness were carried out to measure and evaluate the geopolymers with sodium silicate. The combination of 10 M NaOH with sodium silicate increased the compressive strength by 16.21% and the flexural strength and fracture toughness by 81.6%. However, sodium silicate combined with 12 M NaOH decreased compressive strengths by 13.23% and flexural strength and fracture toughness by 61.94%. The lowest water absorption value of 12.3% was obtained in a geopolymer paste using sodium silicate combined with 10 M NaOH, and the largest was 13.3% for sodium silicate combined with 8 M NaOH. The microstructure analysis showed the hydrated calcium alumina silicate gel (C–A–S–H) and the SEM image also revealed a compact geopolymer matrix. Thus, it can be concluded that sodium silicate from rice husk ash can be utilized as an activator or reactive material to produce geopolymer cement with a good geopolymer network.

## 1. Introduction

Ordinary Portland Cement (OPC) is a construction material that is widely used due to its availability, ease of application, cost-effectiveness, superior mechanical properties, and durability. Currently, cement production is 2.8 billion tonnes yearly and will rise by 4 billion tonnes yearly [1]. This trend shows that global cement demand will grow to 5.5 Gt yearly by 2050 [2]. Its production leads to carbon dioxide (CO_2_) emissions, causing global warming. Cement is an energy-dense material that depletes available natural resources and releases 0.8 tonnes of CO_2_ for every tonne of cement production [3,4].

Several studies have been conducted to find a substitute for cement that is more environmentally friendly and possesses good durability. Many researchers have suggested geopolymers because they are stronger than cement, are more environmentally friendly, and can be produced at lower temperatures [5,6,7]. Geopolymers are a class of “inorganic polymers processed by polycondensation of aluminum and silicon monomeric or oligomeric species in metal alkali-activated solutions” [8]. The geopolymer precursors can be generated from silica and aluminosilicate sources, such as fly ash, metakaolin, clays, etc.

In producing geopolymers, commercial sodium silicate is applied, and they are produced from sand containing high silica and use a high temperature of approximately 1400 °C [9]. An alternative is needed in producing sodium silicate so that it can be more environmentally friendly. Sodium silicate is required by a geopolymer as an activator along with a solution of sodium hydroxide to become an alkaline solution. One alternative material that can be used to produce sodium silicate is rice husk ash (RHA). This material is a reasonably available agricultural waste containing up to 90% SiO_2_, and its application to geopolymers has been reported [10,11]. RHA has been widely examined recently as a promising additive to cement and geopolymer and is also used to produce sodium silicate [12]. However, the mechanical testing of geopolymers on flexural strength and fracture toughness using sodium silicate from RHA has never been carried out.

Using agricultural waste, geopolymer preparation for new applications can be more complicated than using pure materials, because of the impurities in rice husk ash and fly ash. Studies investigating the use of RHA activators in geopolymer applications are limited. Therefore, the chemical explanation of geopolymer formation from industrial byproducts is a challenge. The primary objective of this study was to investigate RHA as a material for making sodium silicate and using it as an activator in geopolymer cement. The monitoring of geopolymer cement formation was characterized by water absorption, bulk density, and compressive strength after 28 days at room temperature. The microstructural properties of at least 28 days of geopolymer cement was determined using X-ray Diffraction (XRD) and Scanning Electron Microscopy Coupled with Energy-Dispersive X-ray (SEM-EDX).

## 2. Materials and Methods

### 2.1. Materials

**Figure 1:**
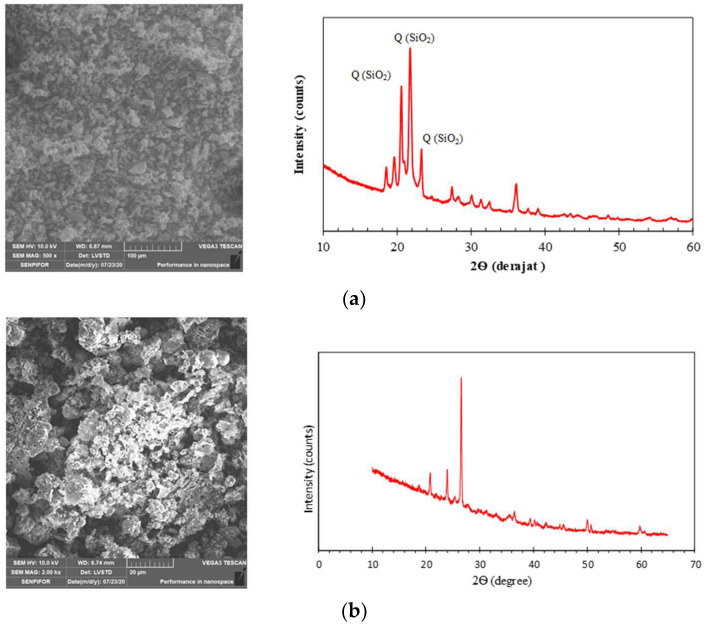
SEM and XRD from fly ash and RHA, (**a**) RHA, (**b**) Fly ash.

This study utilized these materials for synthesizing sodium silicate and industrial by-product-based epoxy-geopolymer pastes. Rice husk ash (RHA) is taken from a refinery in the Aceh Besar District, Aceh, Indonesia. The RHA is white. Table 1 presents the chemical composition of RHA, indicated by XRF, and Figure 1 illustrates the SEM images and XRD of RHA. Class C fly ash (SiO_2_ + Al_2_O_3_ + Fe_2_O_3_ > 70%) (ASTM C618) was taken from the Nagan Raya Thermal Power Project (PLTU Nagan Raya), Aceh, Indonesia. The chemical composition of fly ash was indicated by XRF, as shown in Table 1. The SEM image in Figure 1 shows that the fly ash particles are not spherical and tend to be irregular. From a workability point of view, the fly ash particles with non-spherical particle shapes will increase internal friction, absorption ability, and liquid demand. The fly ash used has an average size of 30 m and an average specific gravity of 2.45. The SEM micrographs obtained for RHA and fly ash are shown in Figure 1, presenting their specific morphologies.

The alkaline solution is the combination of 10 M sodium hydroxide pellets (NaOH) and sodium silicate (Na_2_SiO_3_). The industrial-grade sodium hydroxide pellets, with 98% purity, were dissolved in distilled water. The sodium silicate used in this study is a solution made in the laboratory with the sol –gel method as purposed by some researchers [13,14]. The alkaline solutions in this study for different Na_2_SiO_3_ to NaOH volume ratios were 1:1 and were prepared in the laboratory the day before use. The role of the alkaline solution is to improve the polymerization process with fly ash.

### 2.2. Synthesis of Sodium Silicate

**Figure 2:**
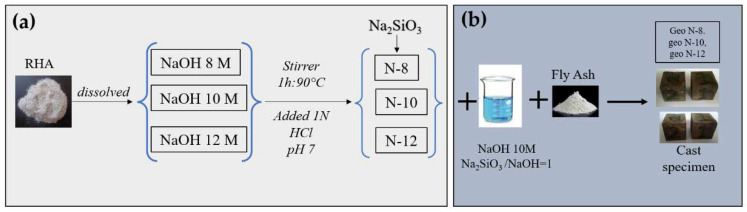
Geopolymer production process, (**a**) synthesis of sodium silicate, and (**b**) preparation of geopolymer paste.

In this study, sodium silicate was prepared in three variations with 8, 10, and 12 M NaOH solutions. Beforehand, NaOH solution was prepared with a molarity of 8, 10, and 12 M. Sodium silicate from RHA was synthesized by attaching the RHA to sodium hydroxide pellets with a ratio of RHA/NaOH solution = 1:6 (*w*/*v*). One hundred grams of rice husk ash was weighed and dissolved in a 600 mL NaOH solution. The assembly was mixed for 1 h at 90 °C under reflux conditions to improve the silica dissolution from RHA, and the mixture was undertaken at 1100 rpm. It was filtered, and then 1 N HCl was added so that the pH of the solution was 7. The gel was then left for 18 h at room temperature. Next, it was washed using warm distilled water and re-filtered. The addition of HCl resulted in a pH of 7 for the solution, and the initial pH of the solution was 12. The addition of HCl, up to pH 7, causes polymerization and the formation of Si-O-Si bonds. This bond will contribute to the geopolymer. The obtained products were nanosilica-based sodium silicates. Specimens prepared with these silicates were denoted as N-X, where X refers to the molarity of the NaOH used in the preparation of sodium silicate. The synthesis of sodium silicate is shown in Figure 2a.

### 2.3. Preparation of Geopolymer Paste

Geopolymer cement pastes were made by placing fly ash into an alkaline solution and mixing for 20 min. Afterwards, the specimens were cast in 5 × 5 × 5 cm^3^ cubes. Specimens were released from the mold after three days. The geopolymer cement specimens, after casting, were placed at ambient temperature (28–30 °C) until the test age of 28 days. Samples are denoted as Geo N-X, where X refers to the molarity of the NaOH used in the preparation of sodium silicate. Table 2 illustrates the mix proportion of geopolymer pastes. The preparation of geopolymer paste is shown in Figure 2b.

### 2.4. Microstructure Characterization

The XRD scans were performed at 10 to 50 °2 Theta with a scan speed of 0.5 s/step. XRD samples were prepared by pressing raw materials between two glass slides into flattening sheets and were analyzed using Shimadzu XRD-7000, Kyoto, Japan. FTIR spectra were measured using Shimadzu-IRPrestige-21, Japan. The spectra were collected in the transmittance mode from a 4 cm^−1^ resolution over a 4000–500 cm^−1^ range. Spectra were recorded at a spectral resolution of 4 cm^−1^, a scan speed of 0.2 cm/s, and were analyzed with Spectrum software.

The microstructures and surface morphologies were tested by the scanning electron microscope (SEM, EVOMA 15, ZEISS, Oberkochen, Germany). The specimens were observed and imaged at an 8 mm working distance and 5 kV accelerating voltage. Sulfur mapping was conducted using EDS adjunct to SEM at a spot size of 5 and magnification of 5000 with a 5 kV accelerating voltage. Synthesized nanosilica-based sodium silicate was measured and analyzed by TEM (JEOL JEM 1400, Peabody, MA, USA). TEM was used to obtain microstructural observations of sodium silicate-based nanosilica with high resolution with a voltage commonly used of 100 kV. The particle size of sodium silicate-based nanosilica was measured using a Particle Size Analyzer (PSA, Horiba SZ-100V2, Kyoto, Japan).

### 2.5. Testing Procedure

#### 2.5.1. Water Absorption, Bulk Density, and Compressive Strength

**Figure 3:**
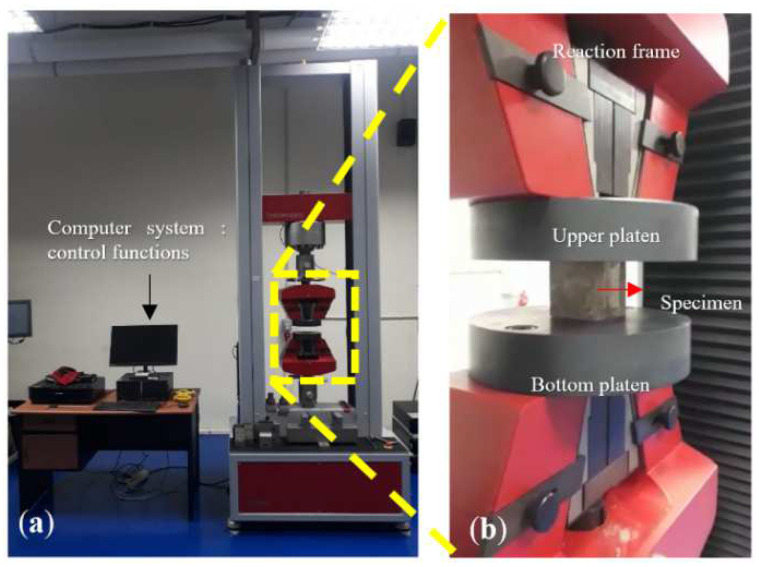
Compressive strength test, (**a**) testing machine, and (**b**) test setup of the specimens.

The water absorption test was conducted following the ASTM C67-07 method. The three specimens were calculated at the average value and were set at 28 days. The bulk density was determined following the ASTM C8300-00 method. The compressive strength test was performed using a testing machine in accordance with ASTM C109. The geopolymer’s compressive strength was calculated after 28 days under the ambient temperature. Three specimens of each synthesized geopolymer cement were examined and reported the average compressive strength values. The testing machine of compressive strength and the test setup are displayed in Figure 3. After the compressive strength test had been carried out, some of the geopolymer fragments were crushed. The powder obtained was used to identify the microstructure such as SEM, XRD, and infrared spectroscopy.

#### 2.5.2. Flexural Strength and Fracture Toughness

**Figure 4:**
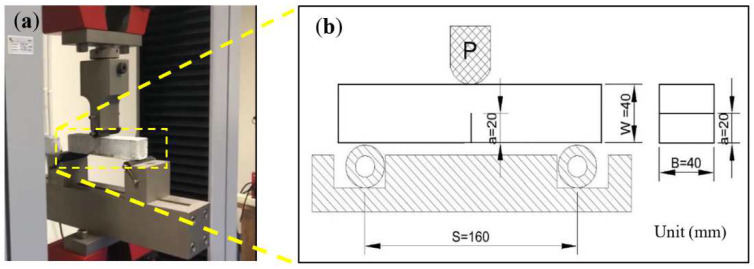
The setup of tools for the three-point bending test: (**a**) setup specimens and (**b**) schematic three bending test [15] with permission.

Three-point bending tests were undertaken to obtain the flexural strength and fracture toughness using Testometric material testing machines with a loading rate of 10 mm/min with a specimen size of 40 mm × 40 mm × 160 mm. At the bottom of the specimens, a crack length (a) of 20 mm was created. A saw was used to trim the specimens. This method was conducted following the ASTM D5045–14 standard. The test setup schematic is shown in Figure 4.

The flexural strength $\left(\sigma_{F}\right)$ was analyzed using the following formula:

(1) $$\sigma_{F}=\frac{3{\;P}_{m}\;S}{2{\;\mathrm{BW}}^{2}}$$

where P_m_ is the maximum load read on the tool, S is the span of the specimen, B is the specimen width, and W is the specimen thickness.

Fracture toughness (K_IC_) was analyzed using the following formula:

(2) $$K_{\mathrm{IC}}=\frac{P_{m}\;S}{{\mathrm{BW}}^{3/2}}\left(\frac{a}{W}\right)$$

(3) $$ƒ\left(\frac{a}{W}\right)=\frac{3{\left(a/W\right)}^{1/2}\left[1.99-\left(a/W\right)\left(1-\;a/W\right)\;\times \;\left(2.15-3.93a/W\;+2.7a^{2\;}/W^{2}\right)\right]}{2\left(1+2a/W\right){\left(1-\;a/W\right)}^{3/2}}$$

where $\left(\frac{a}{W}\right)$ is the ratio of the crack length and the thickness of 0.4, and (a/W), is the polynomial correction factor.

## 3. Results and Discussion

### 3.1. Mechanical Properties of Geopolymer Cement

#### 3.1.1. Bulk Density

**Figure 5:**
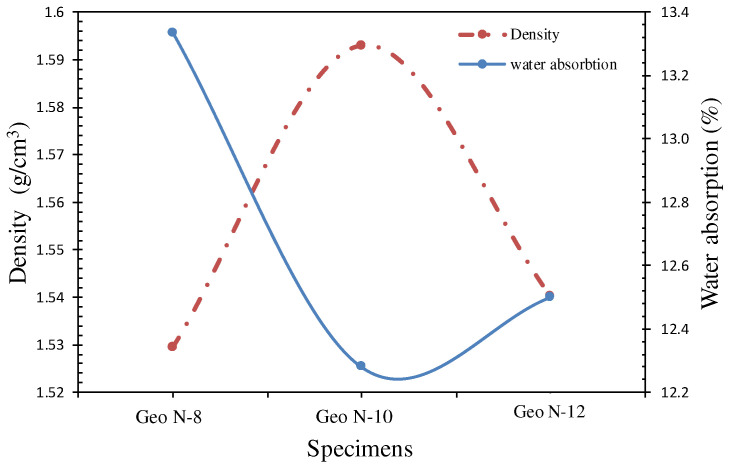
Density and water absorption values with various NaOH concentrations in sodium silicate.

The quality of geopolymer cement can be analyzed by its bulk densities. Geopolymer cement’s bulk density range is from 1.2 to 1.7 g/cm^3^ [16,17]. The bulk density values of specimens containing different sodium silicates are shown in Figure 5. The results indicate that increasing the molarity of NaOH during the sodium silicate synthesis, from 8 M to 10 M, increased the overall bulk density of geopolymer cement. Figure 5 shows that bulk density increased at 10 M NaOH concentration but decreased at 12 M concentration. This may be related to the fact that Geo N-12 paste looked more viscous, which may have made the formation of polymer gels more difficult. The lower bulk density of Geo N-12 geopolymer may also indicate a worse compaction of the specimens with respect to Geo N-10, which may be explained by a higher viscosity of the paste at a fresh state.

#### 3.1.2. Water Absorption

The degree of geopolymerization can lead to a less permeable matrix structure and one that is more dense (less porous). The specimens of Geo N-10 paste presented a minimum water absorption of 12.3% m while the maximum value was obtained at 13.3% at an N-8 sodium silicate concentration. Furthermore, for a given sodium silicate (N-8) value, the geopolymer exhibited a higher water absorption as compared to N-12. This is due to the higher porosity in the specimens and the lower bonding between the fly ash and the geopolymer gel.

Referring to Figure 5, in general, water absorption is reduced with an increase in the density of geopolymer paste. The water absorption value was from 12.3 to 13.3%, under the maximum value of the ASTM-C216 standard (15%). Based on this standard, this material could be used in construction and buildings.

#### 3.1.3. Compressive Strength

**Figure 6:**
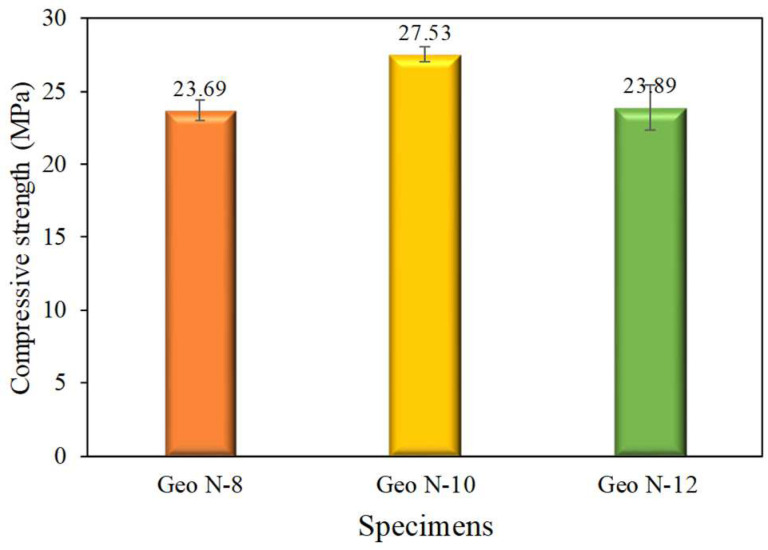
The compressive strength of geopolymer paste.

The critical parameter in the material is the compressive strength that is used to meet engineering quality. Thus, the impact of sodium silicate on the compressive strength of geopolymer cement was decided, and the means are illustrated in Figure 6.

The geopolymer cement’s compressive strength depends on the strength of the geopolymer gel and the interfacial bonding [18]. The results revealed that the compressive strength increased due to the increase in C–S–H and N–A–S–H gels in geopolymer gels formed from sodium silicate in Geo N-10 specimens. Sodium silicate plays a role in the formation of this geopolymer gel. The compressive strength of geopolymers containing sodium silicate with NaOH concentrations of 8 and 10 M reached around 23.69 MPa and 27.53 MPa, respectively. Using the sodium silicate produced with 12 M NaOH concentration, the compressive strength decreased to 23.89 MPa. These results are consistent with studies conducted by previous researchers [13,19,20]. The increase in compressive strength is related to a high amount of silicon dioxide (SiO_2_) in the Geo N-10 specimen. This silica plays a role in the interfacial bonding reaction between the fly ash matrices, thereby increasing its strength [21]. The lower strength was observed for the geopolymer specimens prepared at 12 M NaOH. This can be attributed to the reduced quantity of Si-Al-O bonds.

#### 3.1.4. Compressive Strength and Flexural Strength

**Figure 7:**
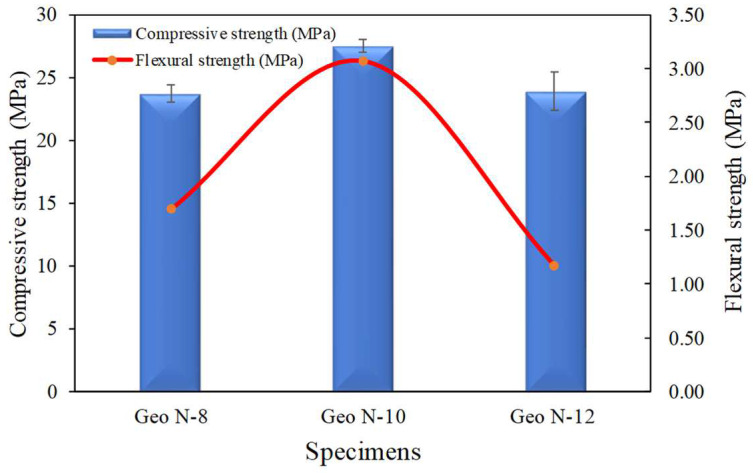
Compressive strength and flexural strength of geopolymer paste.

A similar trend with compressive strength can also be observed regarding flexural strength. The addition of sodium silicate with NaOH molarities of 8, 10, and 12 M resulted in the flexural strength values of 1.69, 3.07, and 1.17 MPa of the geopolymers, respectively. The relationship between compressive strength and flexural strength is shown in Figure 7.

Figure 7 shows an increase in compressive strength by 16.21% for sodium silicate with a molarity of 10 M. In contrast, sodium silicate with a molarity of 12 M reduces the compressive strength by 13.23%. A similar trend also occurs in the flexural strength, which increases in sodium silicate with a molarity of 10 M by 81.61% and decreases in sodium silicate with a molarity of 12 M by 61.94%.

Increasing the molarity of sodium silicate can increase the amount of reaction production of the geopolymer matrix significantly so that the compressive and flexural strength of the geopolymer paste also increases. High alkali and calcium from the fly ash react to form C–H–S, or C–A–S–H and N–A–S–H gels and contribute to the increased mechanical strength of the geopolymer paste [22,23].

#### 3.1.5. Fracture Toughness

**Figure 8:**
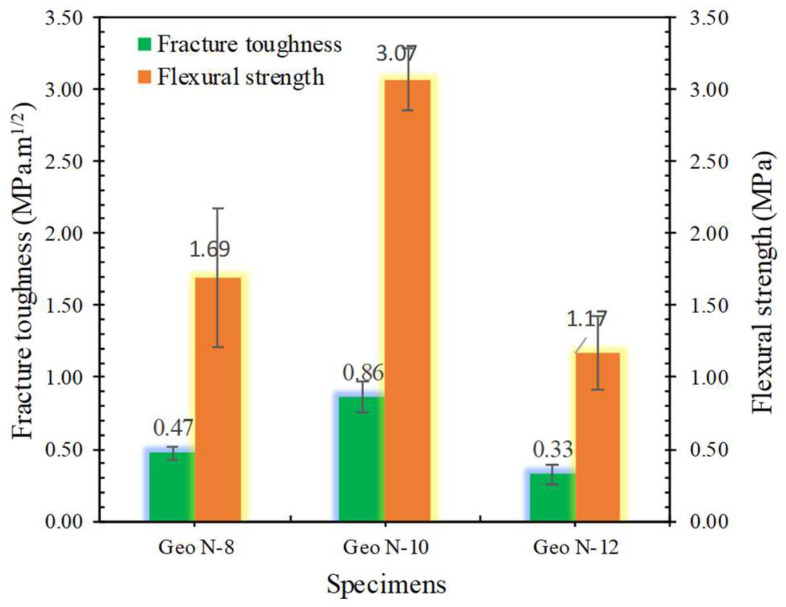
Fracture toughness and flexural strength of geopolymer paste made of various molarities.

Fracture toughness and flexural strength show a similar trend. The magnitude of the fracture toughness of geopolymer pastes with different molarities on sodium silicate and its relationship to flexural strength are shown in Figure 8.

Geopolymers made of sodium silicate with a molarity of 10 M significantly improved the fracture toughness. The sodium silicate with a molarity of 10 M is believed to enhance the mechanical parameters of the gel polymer matrix. Sodium silicate with 8, 10, and 12 M molarity on geopolymer paste resulted in a fracture toughness of 0.47, 0.86, and 0.32 MPa·m^1/2^, respectively. The increase in the fracture toughness of the geopolymer with 10 M sodium silicate is related to the good dispersion and reaction of the silica in the sodium silicate throughout the matrixso as to increase the geopolymer gel and fracture toughness [24].

Table 3 compares the mechanical strength results of this study with several other studies using commercial sodium silicate.

#### 3.1.6. XRD Patterns of Geopolymers

**Figure 9:**
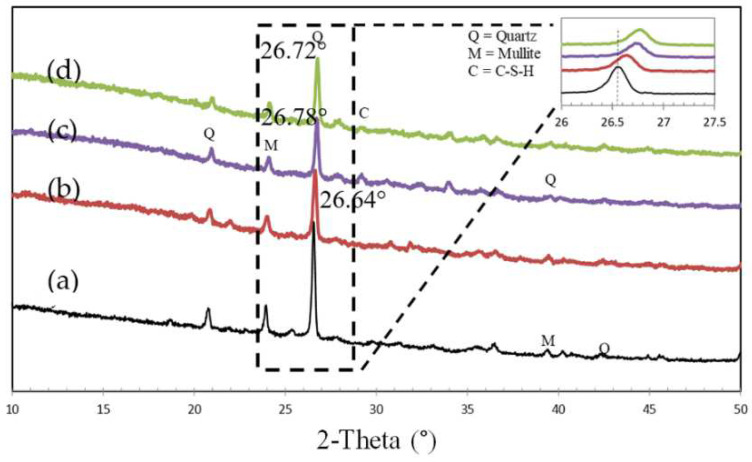
XRD patterns of geopolymer cement: (**a**) fly ash, (**b**) Geo N-8, (**c**) Geo N-10, and (**d**) Geo N-12.

Figure 9 presents the XRD patterns of selected geopolymer paste showing a typical broad hump pattern between 2θ = 25 and 30° centered around 26.64–26.78°2 Theta, which corresponds to the quartz phase that is well-identified in FA.

All XRD patterns show similar behavior. The presence of mineral quartz is observed with the peaks at 26.64°, 26.78°, and 26.72° (2θ, CuKα) in geopolymers with sodium silicate Geo N-8, Geo N-10, and Geo N-12, respectively. The intensity of the X-ray diffraction patterns of the geopolymer with sodium silicate shows nearly similar bands to fly ash (Figure 9a), which is related to the formation of geopolymer networks in all specimens. The intensity of this broad hump is higher on the XRD patterns of sodium silicate with 10 M NaOH (Figure 9c). Mineral phases, mullite, and quartz were visible in the geopolymer XRD patterns, indicating that these minerals remain present during the geopolymerization process. This implies that the higher the SiO_2_/Na_2_O molar ratio, the more sialate (Si–O–Al–O) bonds tend to increase, enhancing the geopolymer network [30].

#### 3.1.7. FTIR Spectra of Geopolymers

**Figure 10:**
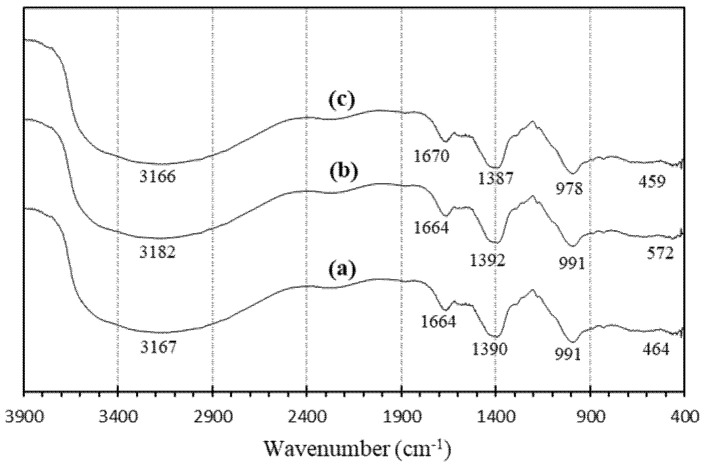
FTIR spectra of geopolymer paste with the sodium silicate from RHA: (**a**) Geo N-8, (**b**) Geo N-10, and (**c**) Geo N-12.

Figure 10 represents the FTIR spectrum of geopolymer paste measured after 28 days.

The FTIR spectrum of the geopolymer pastes showed a band around 464 cm^−1^, ascribed to the Si–O vibration. A very weak band discerned at 459–572 cm^−1^ related to the formation of sialate bonds (Si–O–Al–O) related to the sodium silicate’s polycondensation. This band is characteristic of crystalline cristobalites [31]. The band at 978–991 cm^−1^ indicated that the main geopolymer structure from RHA was Si–O–Al [32,33]. This showed that RHA’s sodium silicate could be used as an activator to produce good geopolymer cement. This band is recognized as the primary band of geopolymer cement and is assigned to Si–O–M (M = Si, Al, Na) [34]. The bands that appeared at 1387–1392 cm^−1^ showed the C–O bond of the carbonate groups. This indicated the emergence of sodium bicarbonate due to atmospheric carbonation. The bending of H–O–H and stretching of (OH) appeared to have formed in the bands at 1664 and 3182 cm^−1^. This signified the presence of bound water molecules in the polymeric framework.

On the specimens of Geo N-8, Geo N-10, and Geo N-12, the value of the wavenumber showed that the asymmetric Si–O–M (M Si, Al, Na, H) almost changed at 1387 cm^−1^. The reduction in this wavenumber that appeared at about 991 and 3182 cm^−1^ in the IR spectrum indicates the depolymerization of the silica network [35].

#### 3.1.8. Microstructure

**Figure 11:**
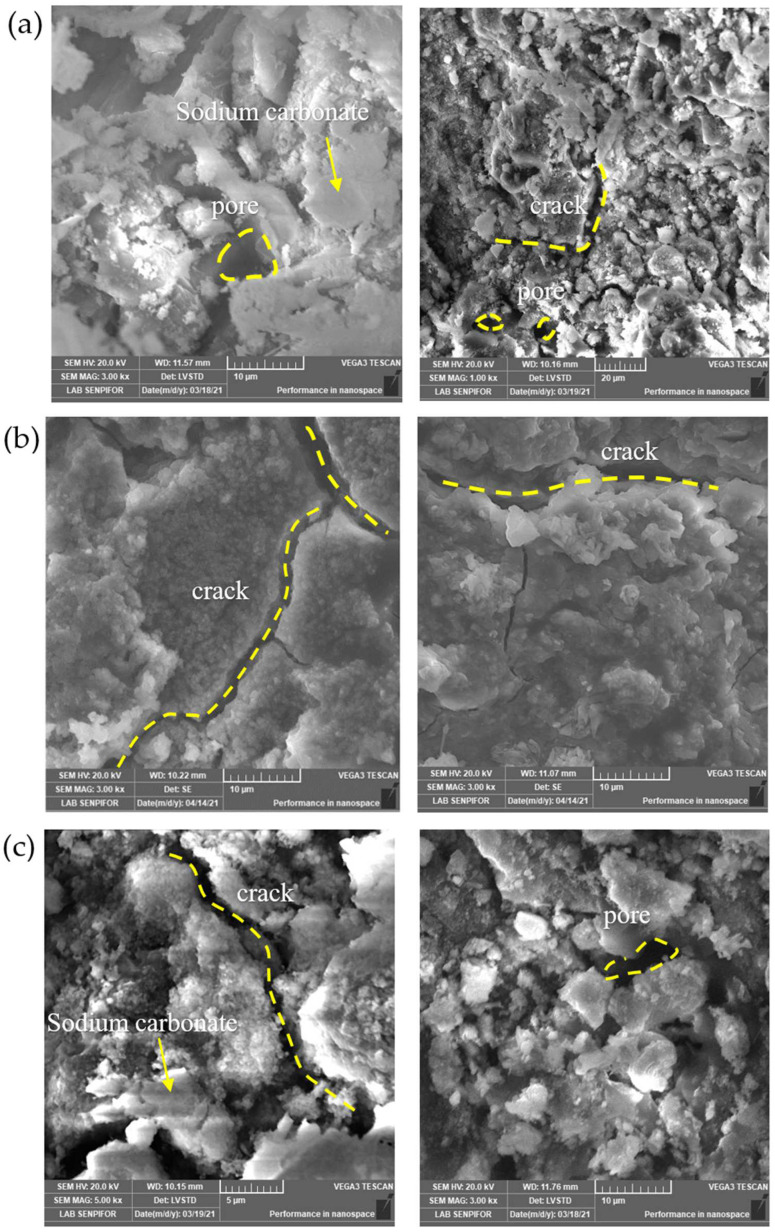
SEM images of geopolymer paste with sodium silicate (**a**) Geo N-8, (**b**) Geo N-10, and (**c**) Geo N-12.

Specimens were analyzed to identify geopolymer paste’s morphological paste obtained with sodium silicate from RHA as an activator. The scanning electron microscope images of geopolymer pastes at 28 days on Geo N-8, Geo N-10, and Geo N-12 specimens are shown in Figure 11.

All images show the presence of micro-fissures related to the previously tested geopolymer paste. The images with higher magnification showed that geopolymer paste has a homogeneous microstructure. Figure 11a,c show the presence of a bright, sponge-like powder indicating that the NaOH present in the alkaline solution did not react to form Na_2_CO_3_. This Na_2_CO_3_-like sponge is commonly found in a NaOH solution-activated geopolymer when excess Na^+^ ions are not bound to the Al^3+^ sites of the geopolymer matrix [20]. This finding is the same as the result of the FTIR analysis in the band 1387–1392 cm^−1^, indicating that the vibration of sodium bicarbonate’s C–O bond is due to atmospheric carbonation.

Micrographs (Figure 11b) did not show much white powder, and the paste appeared to be denser. Thus, Na^+^ ions can bind to Al^3+^ and play a role in improving the mechanical strength of the geopolymer and produce good connectivity between fly ash and sodium silicate solution from rice husk ash. From micrograph investigations, it was observed that sodium silicate made from rice husk ash could be used as an alternative to commercial sodium silicate. Micro-cracks and visible pores appeared in all specimens. RHA dissolved in 10 M NaOH solution showed that the soluble silica formed could play the same role as standard sodium silicate.

## 4. Conclusions

This study showed that silica obtained from an agricultural byproduct can be used to produce sodium silicate and can be applied as an activator for geopolymer cement based on fly ash. The test results demonstrated that the sodium silicate from RHA with NaOH concentrations of 8 and 10 M increased the geopolymers’ density and increased the compressive strength; however, at 12 M, the NaOH concentration in sodium silicate decreases. The best compressive strength and water absorption were at 27.53 MPa and 12.3%. The compressive strength of the geopolymer pastes with NaOH concentrations in sodium silicate 8, 10, and 12 M were 23.69, 27.53, and 23.89 MPa, respectively. The bulk density of the geopolymer based on sodium silicate from NaOH also showed a similar trend. In this study, the best value was found for sodium silicate with 10 M NaOH, namely 1.59 g/cm^3^. In sodium silicate with a 12 M NaOH concentration, there was an increase in the chemical compound Na_2_CO_3_. On specimen Geo N-12, the mechanical strength of the geopolymer paste decreased because the excess Na^+^ was not bounded to Al^3+^ from the geopolymer matrix. The same trend was confirmed in compressive strength, flexural strength, and fracture toughness which each increased by 16.2, 81.6, and 81.6%. The method in this study can be used as an alternative for communities in rice-producing areas to use RHA waste to produce this valuable chemical reagent. The utilization of agricultural waste materials is not only economical but also can lead to environmental pollution control.

## Figures and Tables

**Table 1:** Chemical composition of fly ash and rice husk ash (by weight).

Chemical Composition (%)	Fly Ash	Rice Husk Ash
SiO_2_	21.07	93.27
Fe_2_O_3_	27.23	0.15
CaO	32.58	1.03
Mno	0.44	0.17
K_2_O	1.17	3.41
SO_3_	5.69	0.26
Cl	0.22	0.35
Ag_2_O	0.23	0.10
Al_2_O_3_	9.65	-
TiO_2_	1.68	-
Yb_2_O_3_	0.06	-

**Table 2:** Mix proportion of geopolymer pastes.

Specimens	Fly Ash (g)	NaOH (g)	Na_2_SiO_3_ (g); Was Prepared in Three Variations with	Water (l)	Ratio Na_2_SiO_3_/NaOH
Geo N-8	100	14	35; NaOH 8 M	35	2.5
Geo N-10	100	14	35; NaO 10 M	35	2.5
Geo N-12	100	14	35; NaOH 12 M	35	2.5

**Table 3:** The comparison of mechanical strength across studies.

Reference	Alkaline Activator	Compressive Strength (MPa)	Flexural Strength (MPa)	Water Absorption (%)	Fracture Toughness (MPa·m^1/2^)
This study	NaOH + Na_2_SiO_3_	23.69–27.53	1.17–1.69	12.3–13.3	0.32–0.86
[25]	NaOH + Na_2_SiO_3_	88.0–110.6	2.93–9.32	1.1–7.5	-
[26]	NaOH + K_2_SiO_3_	20–50	0.6–2	-	-
[27]	NaOH + Na_2_SiO_3_	38.3–46.9	4.73–6.63	-	-
[28]	NaOH + Na_2_SiO_3_	-	6.2–7.8	9.9–12.4	-
[24]	NaOH + Na_2_SiO_3_	17.77–22.69	0.91–3.02	-	0.37–1.07
[15]	NaOH + Na_2_SiO_3_	18.16–26.26	0.45–2.62	-	0.16–0.74
[29]	NaOH + Na_2_SiO_3_	50.1–56.7	7.0–9.3	10	-

## Data Availability

Data is contained within the article.
